# The 18th Rocky Mountain Virology Association Meeting

**DOI:** 10.3390/v11010004

**Published:** 2018-12-21

**Authors:** Joel Rovnak, Laura A. St. Clair, Kirsten Krieger, Elena Lian, Rushika Perera, Randall J. Cohrs

**Affiliations:** 1 Department of Microbiology, Immunology and Pathology, Colorado State University, Fort Collins, CO 80523, USA; Joel.Rovnak@colostate.edu; 2 Arthropod-borne and Infectious Diseases Laboratory, Department of Microbiology, Immunology and Pathology, Colorado State University, Fort Collins, CO 80523, USA; stclairl@colostate.edu (L.A.S.C.); Kirsten.Krieger@rams.colostate.edu (K.K.); elian@rams.colostate.edu (E.L.); Rushika.perera@colostate.edu (R.P.); 3 Departments of Neurology and Immunology/Microbiology, University of Colorado School of Medicine, Aurora, CO 80045, USA

**Keywords:** virus, prion, meeting, oligoadenylate synthetase-ribonuclease L (OAS RNAse L)

## Abstract

This autumn, approximately 100 scientists and students from the Rocky Mountain area along with invited speakers attended the 18th annual meeting of the Rocky Mountain Virology Association that was held at the Colorado State University Mountain Campus. The two-day gathering featured 31 talks and 33 posters all of which focused on specific areas of current virology and prion protein research. Since the keynote presentation focused on the oligoadenylate synthetase-ribonuclease L pathway the main area of focus was on host–virus interactions, however other areas of interest included virus vectors, current models of virus infections, prevention and treatment of virus infections, separate sessions on RNA viruses and prion proteins, and a special talk highlighting various attributes of targeted next-generation sequencing. The meeting was held at the peak of the fall Aspen colors surrounded by five mountains >11000 ft (3.3 km) where the secluded campus provided the ideal setting for extended discussions and outdoor exercise. On behalf of the Rocky Mountain Virology Association, this report summarizes 42 selected presentations.

## 1. Introduction

**Figure 1:**
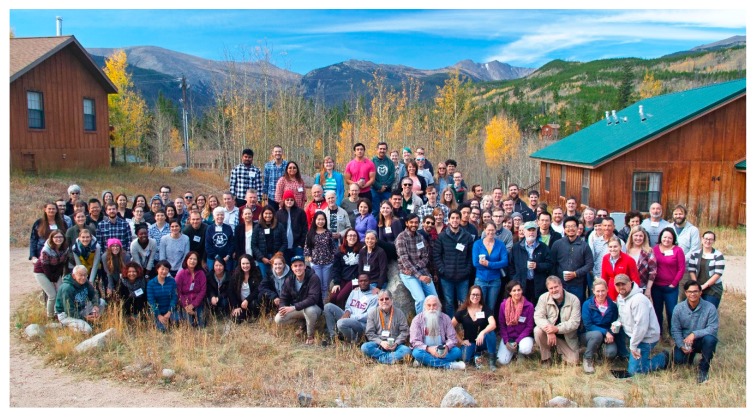
Attendees at the 18th annual Rocky Mountain Virology Association meeting in the Mountain Campus of Colorado State University.

The Rocky Mountain Virology Association was conceived in 2000 to be a regional gathering of virologists where current scientific data is discussed and new ideas shared. In addition, sufficient time was dedicated for graduate students to interact with more established virologists. The venue was selected to be sufficiently removed from urban setting to enhance attendance at all presentations and free time was provided to explore the beautiful mountain setting. Through the years, the meeting has grown to include significant contributions from prion biologists. With many national and international speakers, the meeting has grown past its regional focus, but has still retained its unique ability to provide a forum to advance science and mentorship. Professional childcare was again provided to facilitate attendance by individuals with young children. Not to be outdone, the kids participated in the meeting by showcasing various culinary skills with a virology/prion tilt during the formal poster session. Taken together, the 18th annual Rocky Mountain Virology Association meeting (Figure 1) continued its legacy of virology, prion biology, and mentorship at an altitude of 9000 ft (2.7 km). Selected abstracts are presented below.

## 2. Summary of Scientific Sessions

The meeting began with the keynote presentation by **Susan R. Weiss** (University of Pennsylvania, Philadelphia, PA, USA) who described the antiviral and pro-apoptotic pathway involving oligoadenylate synthetase-ribonuclease L (OAS-RNase L). OAS-RNase L is a potent antiviral pathway that severely limits pathogenesis of many viruses. Upon sensing dsRNA, OASs produce 2′,5′-oligoadenylates (2–5A) that activate RNase L to cleave both host and viral single-stranded RNA thereby limiting protein production, virus replication and spread, and leading to apoptotic cell death. Endogenous cellular dsRNA, which accumulates in the absence of ADAR1, can also activate RNase L and lead to apoptotic cell death. Her team investigated activation and antagonism of RNase L during infections with several types of viruses, especially coronaviruses. They found that RNase L activation in human cells was dependent on OAS3, but independent of virus induced IFN and thus can be activated even in the presence of IFN antagonists. Some betacoronaviruses encode 2′,5′-phosphodiesterases that cleave 2–5A and thereby antagonize activation of RNase. The best characterized of these PDEs is the murine coronavirus (MHV) NS2 accessory protein. MHV-A59 replicates in the CNS causing encephalitis and in the liver causing hepatitis. Enzymatically active NS2 is required for replication in myeloid cells and in the liver, but not in the brain. Interestingly, while wild-type mice clear MHV from the liver by 7–10 days post infection, RNase L knockout mice fail to clear MHV effectively probably due to diminished apoptotic death of infected cells. Dr. Weiss’ team suggested that RNase L antiviral activity stems from direct cleavage of viral genomes, cessation of protein synthesis, and also by promoting death of infected cells, limiting spread of the virus. All animal studies were performed following guidelines and protocols approved by the Institutional Animal Care and Use Committee of the University of Pennsylvania. Funding was provided by R01-AI104887, R21-AI114920, R01-AI110700, and R01-NS081008.

### 2.1. Host–Pathogen Interactions

**Jennifer N. Berger** (Department of Immunology and Microbiology, University of Colorado Anschutz Medical Center, Aurora, CO, USA) investigated how the viral cyclin in murine gammaherpesvirus 68 impacts host and viral gene expression. Several gammaherpesviruses, including the murine model gammaherpesvirus 68 (γHV68), encode a viral cyclin. Previous work in their lab demonstrated that the γHV68 viral cyclin is required for pathogenesis and reactivation from latency. Recently, they demonstrated that expression of *LANA*, a gene critical for maintaining and establishing latency, is dramatically decreased during latent infection in the absence of the viral cyclin. Additionally, lower levels of *LANA* expression in the absence of the viral cyclin are associated with impaired reactivation from latency, even though the number and percentage of infected cells is equal. When viral cyclin deficient latently infected cells are sorted for only *LANA* expressing cells, the defect in reactivation is abrogated, suggesting that latent gene expression primes cells for reactivation. They hypothesize that the viral cyclin coordinates gene expression to facilitate viral reactivation from latency. RNA sequencing and RNA flow cytometry demonstrated subtle differences in host and viral gene expression between cells lytically infected with wild type or viral cyclin deficient viruses. They are currently using single cell RNA sequencing to analyze both permissive fibroblasts and resting B cells for changes in host and viral gene expression following infection with wild type or viral cyclin deficient γHV68. All animal studies were performed following guidelines and protocols approved by the Institutional Animal Care and Use Committee of the University of Colorado-Anschutz Medical Campus.

**Sonja M. Best** (Laboratory of Virology, Rocky Mountain Laboratories, NIAID, NIH, Hamilton, MT, USA) presented data obtained by her postdoctoral fellow, **Abhilash I. Chiramel**, describing new studies on the role of TRIpartite Motif (TRIM) proteins in suppression of flavivirus replication. TRIMs are a large family of proteins, many of which are inducible by type I interferon and serve to suppress virus infection through direct interactions with viral proteins resulting in disruption of viral replication. Primate TRIM5α is a consequential inhibitor that suppresses lentivirus replication (e.g., HIV-1) in a highly host species- and virus species-specific fashion to limit cross-species transmission of these viruses. Importantly, the antiviral effects of TRIM5α are thought to function exclusively in the context of lentivirus infection. Their research interests center on the flaviviruses, which include significant pathogens that have emerged into human populations from primates (e.g., dengue virus, Zika virus, yellow fever virus) prompting them to determine whether TRIM5α could also function to inhibit flavivirus replication. Surprisingly, this work has revealed a new function for TRIM5 as a potent restriction factor for replication of specific flaviviruses. The mechanisms of restriction and its implications for flavivirus emergence and evolution will be discussed. No animal or human studies were performed.

**James M. Burke** (Department of Chemistry and Biochemistry, University of Colorado, Boulder, CO, USA) presented studies that investigated whether ribonuclease L (RNase L) regulates mRNA metabolism, translation, and stress granule assembly in mammalian cells during double-stranded RNA (dsRNA) stress. In response to dsRNA, mammalian cells induce the expression of antiviral genes and arrest translation. The reduction in translation generates RNA-protein (RNP) complexes termed stress granules (SGs), conserved ribonucleoprotein complexes thought to promote the mammalian antiviral response. Both protein kinase R (PKR) and RNase L function to promote translational shut-off. However, while PKR is well-known to promote SG assembly via inhibition of canonical translation initiation, the mechanisms by which RNase L represses translational, whether it contributes to SG assembly, and how antiviral genes are expressed during RNase L-mediated translational arrest has remained unclear. Therefore, Burke and colleagues investigated the effects of RNase L on SG assembly and translation in response to dsRNA. Their studies demonstrated that RNase L promotes rapid and widespread turnover of mRNAs, which limits SG assembly, promotes SG disassembly, and represses translation at early times post-dsRNA. Importantly, the interferon-β (IFN-β) mRNA escapes RNase L-mediated mRNA turnover. This permits translation of IFN-β mRNA at early times post-dsRNA when translational shut-off is primarily driven by mRNA turnover prior to ribosomal RNA (rRNA) degradation. Overall, their findings demonstrate that rapid RNase L-driven translational inhibition is driven by mRNA turnover, which limits SGs and permits RNase L resistant mRNAs, such as (IFN-β) mRNA, to be translated during global host shut-off of translation. No animal or human studies were performed.

**Molly Butler** (Department of Microbiology, Immunology, and Pathology, Colorado State University, Fort Collins, CO, USA) explored the role of cyclin-dependent kinase 8 (CDK8) in regulating dengue-induced metabolic changes. Dengue virus (DENV) is a significant public health concern. There is currently no vaccine available for widespread use and treatment is limited to supportive care. DENV induces glycolysis in infected cells. This upregulation of glycolysis is likely a virally directed mechanism to increase production of metabolic precursors required for particle morphogenesis. She showed that DENV infection results in an upregulation of the expression of CDK8 as well as select glycolytic genes. Increased CDK8 expression increased glycolytic gene expression and infectious particle formation. Inhibition of CDK8 activity with Senexin A reduced glycolytic gene expression during infection. Reduced CDK8 activity did not affect viral genome replication but did reduce infectious particle production. This work suggests that DENV-induced metabolic changes are regulated by CDK8 and are required for infectious particle production. Together, these data provide evidence that CDK8 is a key cellular factor for DENV particle morphogenesis and may serve as a therapeutic target. No animal or human studies were performed.

**Randall J. Cohrs** (University of Colorado School of Medicine, Aurora, CO, USA) discussed the current state herpes simplex virus type 1 (HSV-1) and varicella zoster virus (VZV) transcription and its regulation during latency. HSV-1 and VZV are ubiquitous human neurotropic alphaherpesviruses that establish latency in trigeminal ganglia after primary infection. During primary infection herpesvirus gene transcription follows a well-orchestrated pattern involving immediate-early, early and late stages. However, virus gene transcription is fundamentally different during virus reactivation and involves generalized deregulation of transcriptional controls. Recent targeted RNA and ChIP-seq results obtained from human trigeminal ganglia will be presented that provides missing pieces to the latency puzzle that finally unites VZV and HSV-1. Studies are also presented that describes a culture system to analyze initial events of herpesvirus reactivation. Taken together, the stage is set to address mechanistic questions concerning both HSV-1 and VZV latency/reactivation. No animal or human studies were performed.

**Hannah Jaeger** (Department of Microbiology Biotechnology and Biochemistry, University of Idaho. Moscow, ID, USA) presented her studies on human cytomegalovirus (HCMV), which is known to be the most prevalent cause of neurological birth defects, ranging from microcephaly to sensorineural hearing loss. She elucidated the benefit HCMV derives from modifying a particular cellular mechanism to more efficiently disperse infected cells. Within 6–8 h post infection, HCMV begins to downregulate Nidogen-1 (NID1), an important component of the extracellular matrix (ECM) secreted by endothelial cells, by both protein stability and decreased mRNA transcription. To determine if the absence of NID1 increases dispersal of HCMV, a series of transmigration assays that utilize human umbilical vein endothelial cells (HUVECs) seeded onto a polycarbonate membrane were prepared. THP-1 monocytes were then seeded on top of the HUVEC monolayer and total transmigration of the monocytes was measured after 24 and 48 h. HCMV infection of HUVECS has been shown to increase this transmigration rate, presumably via ECM modifications. Five different treatments of HUVECs, ranging from full HCMV infection to just NID1 knockdown, were used to test the hypothesis that downregulation of NID1 increases transmigration. Preliminary results with uninfected monolayers yielded an average of 24% transmigration vs the absence of NID1 at an average of 35% transmigration. Ultimately, targeting NID1 may provide HCMV a selective advantage, which exacts a negative toll on the developing fetus. This project was supported by a University of Idaho Summer Undergraduate Research Fellowship made possible by a 2017–2018 Undergraduate Research Grant from the Higher Education Research Council/Idaho State Board of Education. They thank Drs. Vic DeFelippis and Ashlee Moses of the VGTI for helpful discussions and provision of reagents for these studies. No animal or human studies were performed.

**Mary McCarthy** and **Tem Morrison** (Department of Immunology & Microbiology, University of Colorado School of Medicine, Aurora, CO, USA) presented work on mechanisms by which chikungunya virus infection disables the functional capacity of the draining lymph node. Chikungunya virus (CHIKV) causes large outbreaks of rheumatologic disease, resulting in chronic disease symptoms in many infected individuals. Studies in humans and animal models suggest that CHIKV infection persists in musculoskeletal tissues, although the mechanisms are unclear. Pathogenic CHIKV strains persist in joints of immunocompetent mice, while the attenuated CHIKV strain 181/25 is cleared by adaptive immunity. The Morrison lab analyzed the draining lymph node (dLN) to define events in lymphoid tissue that may contribute to CHIKV persistence or clearance. Infection with acutely cleared CHIKV resulted in robust dLN enlargement and germinal center (GC) formation. In contrast, the dLN of mice infected with pathogenic CHIKV displayed architectural disruption, with paracortical relocalization of B cells and loss of a distinct B-T border. Lymphocytes were depleted from the dLN during pathogenic CHIKV infection, which was associated with impaired expansion and function of high endothelial venules. Lymphocyte depletion and disorganization was preceded by infiltration of the dLN with myeloid cells early after pathogenic CHIKV infection. Preventing the early influx of inflammatory monocytes, but not neutrophils, to the dLN reversed depletion of dLN B cells in a manner partially dependent on production of either nitric oxide or superoxide. Furthermore, monocyte depletion improved dLN GC formation and CHIKV-specific serum neutralizing antibody responses. These data suggest that the rapid influx of inflammatory monocytes to the dLN triggered by pathogenic, persistent strains of CHIKV impairs the development of adaptive immune responses, providing new insight into the immunologic mechanisms that facilitate CHIKV persistence in musculoskeletal tissue. All animal studies were performed following guidelines and protocols approved by the Institutional Animal Care and Use Committee at the University of Colorado School of Medicine (Assurance Number A3269-01). Funding: U19 AI109680, R01 AI108725, and F32 AI122463 from NIH-NIAID.

**Daniel Michalski** (Department of Microbiology, Immunology and Pathology, Colorado State University, Fort Collins, CO, USA) presented data demonstrating how the generation and accumulation of ZIKV sfRNA during infection compromises RNA metabolism by targeting host cell factors involved in multiple pathways. Zika virus (ZIKV), a single-stranded positive sense RNA flavivirus transmitted primarily by *Aedes aegypti* (mosquito), generates stable subgenomic flavivirus RNAs (sfRNA) due to the stalling of the major cytoplasmic 5′–3′ exoribonuclease XRN1 at a knot-like three helix junction structure located in viral 3′ untranslated region (UTR). sfRNA decay intermediates accumulate to high levels in infected cells and studies with other flaviviruses have implicated sfRNAs in cytopathology. Their lab’s objective is to characterize the function of ZIKV sfRNAs to gain insight into ZIKV pathogenesis. Specifically, they focused on two key aspects: First, they determined the impact of ZIKV sfRNA generation and accumulation on mRNA abundance and stability. Second, they identified host proteins that interact with ZIKV sfRNA and have begun to evaluate their role in cytopathology/pathogenesis. Infection of JAR cells with WT ZIKV showed a significant increase in both abundance and stability of cellular mRNAs which correlated with sfRNA accumulation over the time course of infection. RNA pull-down experiments revealed that DDX6, a translational control and deadenylation-dependent mRNA decay factor, binds to ZIKV sfRNA. Additional host cell RNA binding proteins (RBPs) involved in mRNA translation and decay, including EDC3 and LSM14B also interact with ZIKV sfRNA. Furthermore, many other key RBPs involved in RNA processing were identified and prioritized for further study. Thus, in addition to targeting XRN1, sfRNAs appear to interact with a ‘regulon’ of RBPs to disrupt cellular mRNA decay regulation as well as other RNA processing factors in an effort to compromise multiple steps of RNA metabolism and promote pathogenesis. No animal or human studies were performed.

**Zoe O’Donoghue** (University of Colorado, School of Medicine, Aurora, CO, USA) discussed data indicating that there are multiple classes of RNA secondary structures present in flaviviral 3′UTRs which nonetheless still serve the same function during viral infection. Flaviviruses such as yellow fever, dengue, West Nile, and Zika generate disease-linked viral non-coding RNAs called subgenomic flavivirus RNAs (sfRNAs). sfRNAs have been implicated in a variety of interactions with both the host and vector during infection and have been shown to be necessary for cytopathicity and pathogenicity. sfRNAs accumulate as the 5′ to 3′ progression of cellular exoribonuclease Xrn1 is blocked by structured RNA elements called Xrn1-resistant RNAs (xrRNAs) located within the viral genome’s 3′UTR that operate without protein co-factors. Here, they directly demonstrate that xrRNAs, and the ability to produce sfRNAs, exist in a diverse set of flaviviruses, including some specific to insects or with no known arthropod vector. SHAPE chemical probing data indicate that these xrRNAs comprise two secondary structural classes that align with previously reported phylogenic analysis. These discoveries have implications for the evolution of exoribonuclease resistance, the use of xrRNAs in synthetic biology, and for the development of targeted therapies to combat flavivirus diseases worldwide. No animal or human studies were performed.

**Amber Rico** (University of Nebraska–Lincoln, School of Veterinary Medicine and Biomedical Sciences, Lincoln, NE, USA) presented work describing vaccinia virus usurpation of host cellular genes. The vaccinia B1 kinase is an essential viral protein that promotes vaccinia infection at multiple junctures by interacting with and subverting host cellular pathways. To elucidate the mechanisms through which B1 interacts with these cellular pathways, they studied vaccinia replication in the absence of B1. These studies identified cellular VRK2 as an upstream regulator of vaccinia DNA replication. Using immunofluorescence of replication factories and DNA replication assays they characterized the replication block caused by B1 and/or VRK2 deletion. VRK2 deletion alone did not affect wild-type vaccinia replication; whereas, the deletion of B1 alone delayed but did not block vaccinia DNA replication. Importantly, deletion of both B1 and VRK2 resulted in a lasting vaccinia DNA replication block. Their current data suggests that VRK2 functions to phosphorylate a yet unidentified substrate which rescues vaccinia replication in the absence of B1. B1 and VRK2 have been implicated in the phosphorylation-dependent regulation of several cellular processes including apoptosis, translation, protein degradation, and intrinsic immunity in previous publications. However, despite attempts to connect previously identified substrates of B1 or VRK2 with the VRK2 dependent phenotype, they have been unsuccessful. The present study has highlighted the utility of using vaccinia deletion viruses for the identification of virus–host interactions. Using a B1-deletion virus, they characterized a novel role for B1/VRK2 during the very early stages of vaccinia viral replication. Ongoing studies are aimed at identifying both the substrate and mechanism through which VRK2 promotes vaccinia replication in the absence of B1. No animal or human studies were performed.

**Scott Seitz** (Department of Immunology and Microbiology and the Department of Neurology, University of Colorado Anschutz, Aurora, CO, USA) explored the effects of pharmacologic depletion of microglia and how it increases mortality in a flaviviral model of murine encephalitis. Microglia are the resident immune cells of the central nervous system (CNS) and maybe critical in controlling neuroinflammatory responses during viral infection. Microglia help to facilitate viral clearance by active phagocytosis of infected material, secretion of antiviral cytokines and chemokines, and maintenance of the blood brain barrier. Flaviviruses represent a wide range of viruses that cause neurological disease. The role of microglia during flavivirus-induced CNS disease is poorly understood. Microglia are dependent on colony-stimulating factor 1 receptor (CSF1R) signaling for their survival. They used an inhibitor of CSF1R signaling to investigate the role of microglia in response to two flaviviral infections: West Nile virus (WNV) and Japanese encephalitis virus (JEV). They showed that treatment of mice with PLX5562 depleted the majority of microglia from the CNS (>80%). Depletion of microglia altered cytokine and chemokine responses during viral infection and was associated with increased mortality (up to 100% with WNV), earlier onset of disease and increased viral loads within the CNS. This study and further studies help to suggest that (i) microglia play an important role in surviving flaviviral induced encephalitis; (ii) lack of microglia leads to increased viral burden within the CNS of infected animals; and (iii) production of many chemokines and cytokines are not solely secreted by microglia. All animal studies were performed following guidelines and protocols approved by the Institutional Animal Care and Use Committee of the University of Colorado Anschutz.

**Obaiah Dirasantha** (University of Colorado at Boulder, BioFrontiers institute, Department of Molecular Cellular and Developmental Biology, CO, USA) presented data describing the interaction between pandemic HIV-1 and its host receptors, CD4 and CCR5, within a population of captive owl monkeys. The HIV-1 surface protein, Envelope (Env), mediates interaction with the CD4 and CCR5 receptors of T cells for attachment and entry. Their lab and others have recently demonstrated that most primate CD4 molecules are incompatible with pandemic (group M) HIV-1. This presents a major obstacle for modeling HIV-1 in nonhuman primates. In contrast, they recently showed that Central and South American owl monkeys encode a CD4 receptor that is compatible with pandemic HIV-1. This makes owl monkeys of interest as a possible new animal model for HIV-1 pathogenesis and transmission. Based on their previous findings of CD4 polymorphism within this species, they are now undertaking a systematic study of receptor polymorphism within these animals. In this study, they genotyped CD4 and CCR5 from 163 owl monkeys (*Aotus nancymaae*). They identified numerous CD4 (*n* = 11) and CCR5 (*n* = 2) receptor alleles that encode unique protein variants. They then generated stable cell lines expressing all possible CD4/CCR5 receptor combinations and challenged them with various forms of pandemic HIV-1. Likewise, they are currently screening over 52 forms of pandemic HIV-1 Env for those that are compatible with owl monkey CD4/CCR5 receptors. The identification of owl monkeys or particular Envs that support HIV-1 infection has tremendous potential in improving our ability to model HIV-1 infection in nonhuman primates. All animal studies were performed following guidelines and protocols approved by the Institutional Animal Care and Use Committee of Department of Veterinary Sciences, Michale E. Keeling Center for Comparative Medicine and Research, University of Texas MD Anderson Cancer Center.

**Emily R. Feldman** (Department of Molecular, Cellular, and Developmental Biology, BioFrontiers Institute, University of Colorado at Boulder, Boulder, CO, USA) investigated functional diversity of the HIV-1 restriction factor tetherin within a captive population of owl monkeys. Restriction factors are intracellular proteins that inhibit viruses at numerous stages of their replication cycle. Primate lentiviruses, including simian immunodeficiency viruses (SIVs) and HIV-1, have evolved numerous countermeasures to combat restriction factors. However, these are highly host specific, and in many cases, are ineffective when confronting a new host species. For instance, rhesus macaque Tetherin is highly effective at blocking HIV-1 infection, while the human version is not. In fact, the macaque versions of most restriction factors block HIV-1 replication (Tetherin, TRIM5a, SAMHD1, MX2, and APOBEC3s). This is problematic because HIV-1 vaccine testing has mainly been performed in macaques. Owl monkeys, on the other hand, are of significant interest because they are the only known monkey species that encode minimal blocks to HIV-1 infection, including a functional HIV-1 entry receptor (CD4). Previous studies have identified non-functional Tetherin alleles in owl monkeys. Based on this, they are now undertaking a systematic study of Tetherin polymorphism in other captive owl monkey species. They have genotyped Tetherin from 45 owl monkeys (*Aotus nancymaae*) and have identified eight alleles that encode unique protein variants. They have generated stable cell lines expressing tetherin alleles and challenged them with various HIV-1 isolates. In addition, they are currently screening HIV-1 and SIV *Nef* genes for their ability to counteract owl monkey tetherin. The identification of owl monkeys with defective tetherin alleles, or particular *Nef* versions that efficiently degrade owl monkey Tetherin, has tremendous potential in furthering owl monkeys as novel nonhuman primate models of HIV-1 infection. No animal or human studies were performed.

**Conor Kelly** and **Edward Chuong** (BioFrontiers Institute, University of Colorado Boulder, Boulder, CO, USA) presented preliminary data exploring the possible involvement of species-specific transposable elements in the evolution of the type II interferon response. Endogenous retroviruses (ERVs) constitute 6–14% of mammalian genomes and contain transcription factor-bound regulatory sequences. Functional genomic studies implicate ancient ERVs as a source of regulatory elements that have been evolutionarily co-opted to regulate processes such as immunity and development. However, given that ERVs are highly lineage-specific, this raises the question of how ERV co-option in different species may shape their biological differences. The Chuong lab previously identified primate-specific ERVs that have been co-opted as enhancers to regulate interferon-inducible innate immune responses in human cells, by providing binding sites for the transcription factor STAT1. A pressing question is whether the responses in other mammalian species have also been shaped by ERV co-option. To investigate this idea, they are analyzing the genome-wide responses to interferon gamma (IFNG) in cells from non-primate species, namely mouse (*M. musculus*), dog (*C. l. familiaris*), and cow (*B. taurus*). Their preliminary analysis of published mouse chromatin profiling and transcriptomic datasets assaying bone marrow-derived macrophages has revealed that, out of 5,507 STAT1 binding events in IFNG treated cells, 1,165 (21%) of these sites were derived from ERVs and other transposons. 446 of these transposon-derived STAT1 sites were located within 50 kilobases of interferon-stimulated genes. They selected 50 candidate enhancers derived from ERVs and other transposons that potentially regulate mouse interferon-stimulated genes—including *Dicer1*, *Casp8*, and *Pycard*—which they will test for regulatory function by using CRISPR-based approaches in mouse macrophage cell lines. The Chuong lab’s preliminary results suggest that primate and rodent immune responses have undergone evolutionary rewiring, facilitated by lineage-specific ERVs. No animal or human studies were performed.

**Ashley Knox** (Department of Microbiology and Immunology, University of Colorado, School of Medicine, Aurora, CO, USA) presented data examining the effect of viral infection on the transcription of both host and viral non-coding RNAs. While viral RNA is known to play vital roles in viral infection and replication, the regulation of viral RNA polymerase III (Pol III)-transcribed non-coding RNAs (ncRNAs) remains unclear. The gammaherpesviruses (γHVs) contain multiple ncRNAs that are highly expressed during infection. The γHV68 TMER ncRNAs are required for pathogenesis and Epstein–Barr virus ncRNAs (EBERs) contribute to B cell transformation. Additionally, several host ncRNAs have been shown to be upregulated during gammaherpesvirus infection and play integral roles in pathogenesis. These include the B2 SINEs, which stimulate NF-κB signaling that ultimately increases viral gene expression, and vault RNAs that protect B cells from apoptosis and allow enhanced viral establishment. Therefore, detailing how these ncRNAs are regulated is integral to understanding their role in pathogenesis. To compare the transcriptional regulation of Pol III ncRNAs, they cloned several types of Pol III promoters into a newly generated luciferase reporter they have optimized to measure Pol III promoter activity during infection. Promoters include those of host ncRNAs (5S rRNA, tRNA, U6 snRNA), and of the γHV68 TMERs, EBV EBERs, and adenovirus VA RNA. Infection with γHV68 drives simultaneous repression of the human U6 promoter activity and stimulation of the γHV68 TMER promoters. This TMER promoter induction was prevented by an inhibitor of viral protein synthesis, indicating that this aspect of infection is vital for driving transcriptional regulation. This analysis of Pol III promoter activity indicates unique regulation patterns for host and viral ncRNAs, and identifies the critical promoter features for preferential expression during infection. No animal or human studies were performed.

**Kirsten Krieger** (Arthropod-borne and Infectious Diseases Laboratory, Department of Microbiology, Immunology and Pathology, Colorado State University, Fort Collins, CO, USA) presented data on using the CCTalpha enzyme as a control point to limit dengue virus serotype 2 replication. Causing over 390 million cases of dengue fever annually, dengue viruses (DENV) are the most aggressive arthropod-borne human pathogens. A characteristic of many positive-sense RNA viruses is the shared ability to restructure host cell lipid membranes in order to assemble viral replication complexes. These viruses reorganize host lipids to benefit themselves in constructing platforms for their replication and assembly. Phosphatidylcholine (PC) is an amphipathic lipid which makes up about 50% of the total phospholipid content in eukaryotic cellular membranes. Previous studies on different positive-sense RNA viruses have shown PC accumulation at viral replication sites in cells infected with brome mosaic virus, hepatitis C, and poliovirus. They have shown that PC synthesis is significantly enhanced during DENV-infection of mosquito cells. PC is synthesized via the Kennedy pathway or the PEMT pathway. The PEMT pathway is only common in liver cells where it produces maximum 30% PC, however the Kennedy pathway is present in all nucleated eukaryotic cells and is abundant in most tissues. The rate-limiting step of PC synthesis is catalyzed by the CCTalpha enzyme. siRNA knockdown of CCTalpha decreases DENV replication. Knocking down over 65% of CCTalpha, her lab has also shown that the siRNA used is not cytotoxic to Huh-7 cells. Her lab is validating the role of this enzyme under the hypothesis that CCTalpha is used by DENV at viral replication sites by amplifying PC synthesis locally and thus changing the lipid composition of membranes to benefit the viral life cycle. No animal or human studies were performed.

**Elena Lian** (Arthropod-borne and Infectious Diseases Laboratory, Department of Microbiology, Immunology and Pathology, Colorado State University, Fort Collins, CO, USA) presented preliminary data to evaluate arachidonic acid as a mediator of membrane biogenesis during dengue virus-serotype 2 infection. Dengue viruses are attributed with an estimated 400 million annual infections and is endemic in the tropical and subtropical regions of the world. These viruses replicate on the membrane of the endoplasmic reticulum (ER) and alter the host’s lipid biosynthetic pathways to achieve this purpose. Of interest is arachidonic acid (AA), a long polyunsaturated fatty acid that can be incorporated into cellular membranes to increase membrane fluidity, and is also the progenitor of leukotrienes that act as signaling molecules. Fatty acid desaturase 2 (FADS2) is the rate-limiting enzyme of the linoleic acid pathway that synthesizes AA as a metabolic intermediate. Downstream of AA is 5-lipoxygenase (5-LOX) catalyzing the production of leukotrienes, one of which has been shown to interact with fatty acid synthase (FAS). FAS, as the key enzyme in de novo fatty acid synthesis, produces long chain fatty acids that can also be integrated into membranes. Her lab hypothesizes AA-mediated membrane biogenesis facilitates the formation of viral replication complexes on the ER membrane. Chemical inhibition and siRNA analyses have been used by her lab to target FADS2 and 5-LOX to elucidate the role of AA during infection by dengue virus-serotype 2 (DENV2). Preliminary results indicate a decrease in viral release when either enzyme is inhibited during DENV2 infection, thus presenting FADS2 and 5-LOX as two enzymatic controls to limit viral replication and transmission. No animal or human studies were performed.

**María Mora Gonzalez Lopez Ledesma** (Laboratorio de Virología Molecular, Fundación Instituto Leloir-CONICET, Buenos Aires, Argentina) assessed protein–protein interactions between the viral protein NS5 of dengue virus and cellular host proteins and extended those studies to Zika virus infection. Dengue virus (DENV) is one of the most important human viral pathogens transmitted by insects; it belongs to the Flaviviridae family, together with other human pathogens, such as West Nile virus, Japanese encephalitis virus, and Zika virus (ZIKV). The recent outbreak of ZIKV in America and its link to microcephaly put this virus at the forefront of an international research effort to understand its cellular targets and pathogenesis. Flavivirus NS5 protein plays multiple functions during infection, enabling viral replication and counteracting host antiviral responses. To investigate the interaction of NS5 with cellular components, they performed proteomic studies using an NS5 tagged DENV. They identified 53 host proteins that bind or form complexes with NS5 during infection and validated a number of these interactions. Among the NS5 binders, they found core components of the host spliceosome machinery: components of the U5 snRNP particle (CD2BP2, DDX23, and EFTUD2). Interestingly, silencing of these proteins enhanced DENV viral replication. A model was proposed, in which NS5 binding to U5 snRNP proteins hijacks the splicing machinery resulting in a less restrictive environment for viral replication. These studies were extended to ZIKV NS5; they have recently observed that down-regulation of CD2BP2 slightly increased ZIKV replication, although further studies are needed. In summary, a technology to investigate protein–protein interactions during viral infection was developed and was used to define novel functions of the viral protein NS5. The vast amount of information provided by this study is currently being used to validate protein–protein interactions in DENV and ZIKV infection. No animal or human studies were performed.

**Caroline Montgomery** and **Gaby Ramirez** (Department of Microbiology, Immunology and Pathology, Colorado State University, Fort Collins, CO, USA) explored dengue viruses (DENV), members of the Flaviviridae family that are transmitted by *Aedes aegypti* mosquitoes infecting over 400 million people annually. There are no antivirals and only a suboptimal vaccine available for protection. DENV manipulates the lipid metabolic pathways of the host to acquire a lipid envelope, with the purpose to assemble membranes for genome replication and for immune evasion. They are identifying novel targets for antiviral development by understanding how these viruses hijack host lipid metabolic pathways for their benefit. The goal is to inhibit metabolic enzymes required for virus replication and choke acute virus infection in the human host. They have shown that the unsaturated fatty acid biosynthesis pathway is manipulated by DENV. These fatty acids maintain membrane fluidity and can be incorporated into complex lipids. Stearoyl Co-A desaturase (SCD1), a rate-limiting enzyme in the homeostasis of unsaturated fatty acids, is increased upon infection with DENV. They have now assessed the significance of enzymes downstream of SCD1 in the DENV life cycle. These enzymes include glycerol-3-phosphate acyltransferase (GPAT), acylglycerolphosphate acyltransferase (AGPAT), diacylglycerol acyltransferase (DGAT), and Acyl-CoA:cholesterol acyltransferases (ACAT). They hypothesize that DENV replication will be reduced by inhibiting enzymes downstream of SCD1, leading to a lower availability of UFAs required for genome replication and enveloped particle assembly. They have carried out loss of function studies on these enzymes and determined their impact on DENV replication. Preliminary results will be presented. No animal or human studies were performed.

### 2.2. Vector Studies

**Taylor Clarkson** (Colorado State University, Fort Collins CO, USA) presented data exploring nootkatone, a natural organic compound in grapefruit and Alaskan yellow cedar, as a novel insecticide and insect repellent. They tested a nootkatone product for insecticide and repellency activity against the two most prominent vectors of Zika virus (ZIKV), *Aedes aegypti* and *Aedes albopictus*. They both tested pyrethroid-resistant and pyrethroid-susceptible strains of these species to determine whether or not this physiological status affected their susceptibility to nootkatone, and they conducted their assays using both uninfected and ZIKV-infected mosquitoes. CDC bottle bioassays showed the pyrethroid-susceptible strains of both *Ae. aegypti* and *Ae. albopictus* were more susceptible to nootkatone then the pyrethroid-resistant strains. These assays were also conducted with ZIKV-infected mosquitoes as well, showing that ZIKV-infected mosquitoes were significantly more susceptible to nootkatone than uninfected controls. The repellency and biting inhibition of nootkatone was primary tested on treated human arms using a repellency/irritancy and biting inhibition bioassay (RIBB). In these bioassays, three different test subjects tested various types of existing repellency products, and several concentrations of the nootkatone formulations applied on one of their arms, and the spatial attractive index and biting inhibition of *Ae. aegypti* were measured. In general, their conclusions discovered that nootkatone worked to repel mosquitoes at a rate comparable to 7% DEET or 5% Picaridin, and has the potential to be an efficacious insecticide against *Aedes* mosquitoes, both ZIKV-infected and uninfected. Funding from EVOLVA and the NIH. All studies using human subjects or tissue samples have been either approved or deemed non-human subject research by the Institutional Review Board of Colorado State University.

**Daniel Hartman** (Colorado State University, Department of Microbiology, Immunology, and Pathology, Fort Collins, Colorado, USA) presented entomological work exploring the risk that Rift Valley Fever Virus introduction may pose to feed lots. If introduced, RVFV could cause significant economic losses to the livestock industry in addition to substantial human morbidity and mortality, and likely establish itself as a zoonosis with little hope of eradication. To explore the potential for transmission of RVFV in agricultural settings that support intensive livestock production, the Kading Laboratory investigated the mosquito community composition at livestock feedlots and surrounding natural and residential areas. *Culex tarsalis* Coquillett mosquitoes were hypothesized to be locally-important bridge vectors in the event of an introduction of RVFV due to their abundance, competency for transmission of RVFV, and seasonal fluctuations in blood host selection that includes large mammals and humans. Mosquitoes were trapped using a paired-site design with traps placed at feedlots, as well as nearby sites to determine whether the presence of livestock affected mosquito abundances and community composition. Significant associations were noted between mosquito species and habitat type. *Cx. tarsalis* was collected on both feedlots and nearby sites, and diverse vertebrate blood meals were detected, including cattle. These data indicate a propensity for *Cx. tarsalis* to feed on cattle, and also suggest dispersal of this species between feedlots and the surrounding areas. These data support a potential for *Cx. tarsalis* to serve as a bridge vector of RVFV between human and livestock communities in Colorado. No animal or human studies were performed.

**Ashley Janich**, along with **Karla Saavedra-Rodriguez** and **William Black IV** (Department of Microbiology, Immunology and Pathology, Colorado State University, Fort Collins, CO, USA) investigated aspects of permethrin resistance in *Aedes albopictus* mosquitoes from Southern Mexico. Insecticide resistance and its known mechanisms have been extensively studied in *Aedes aegypti* mosquitoes, the major vectors of Zika, dengue, and chikungunya viruses. However, another important vector is *Aedes albopictus*. The primary method to control the spread of these vectors and their diseases is by insecticide application. With continued application of insecticides like permethrin, *Ae. aegypti* has developed an observable resistance, requiring increasingly higher concentrations to be killed. It is important to understand if *Ae. albopictus* is also developing resistance and what possible mechanisms are involved. They collected *Ae. albopictus* from Chiapas, MX, reared them in their lab, and exposed adults from each location to several concentrations of permethrin. The concentration needed to kill 50% of the mosquitoes (LC_50_) and resistance ratios (RR) were calculated. Current results show lower mortality in mosquitoes from Mexico compared to the control, with RR ranging from 1.27 to 2.06, indicating there is a low level of resistance. They predict to see an increase in the RR with continuous selection of survivors and their offspring (results pending). Additionally, they will sequence and screen DNA from mosquitoes that survive permethrin exposure for known resistance polymorphisms to see if there are mutations that are conferring this resistance. The results of this study will be helpful when considering how to proceed with future methods of mosquito control. Funding: NIH Grant (1R01AI121211-01A1) “Insecticide Resistance Management to Preserve Pyrethroid in *Aedes aegypti*.” No animal or human studies were performed.

### 2.3. Modelling Disease

**Amy Gilbert** (National Wildlife Research Center, USDA APHIS Wildlife Services, Fort Collins, CO, USA) described the epidemiology of rabies virus (RABV) infections in wildlife in the United States, and research investigating recent epizootics of the south-central skunk RABV lineage in northern Colorado and southern Wyoming. Rabies is a highly lethal zoonosis, and RABV circulation is maintained by a diverse number of bat and wild carnivore reservoirs in North America. Lineages circulating in carnivores cause a greater burden of spillover infections in animals and exposures to humans, especially where RABV circulates in raccoons (*Procyon lotor*). Striped skunks (*Mephitis mephitis*) have been a recognized RABV reservoir in North America for much of the past century, and several lineages recognized decades ago are still circulating today. The south-central skunk (SCSK) lineage is one historic lineage that has expanded its geographic range. Colorado has typically been on the periphery of SCSK circulation, but after decades of apparent absence, the lineage re-emerged in 2007 and two significant epizootics were detected in northern Colorado staring in 2012. This study analyzed case data collected by local public health departments in northern Colorado during 2012–2015, and enhanced surveillance starting in 2013, to describe the spatio-temporal spread of the epizootics. As a result of enhanced surveillance and collaborative testing effort, their study also opportunistically documented other wild carnivore pathogen infections, including Bartonella and canine distemper virus. The results are discussed in light of the implications for human and animal health. No animal or human studies were performed.

**Keir M. Balla** (Department of Human Genetics, University of Utah School of Medicine, Salt Lake City, UT, USA) intended to describe efforts to develop zebrafish as a model for virus host switching and evolution of the vertebrate immune system. Recent large-scale genomic and phylogenetic studies have revealed that host switching is extremely pervasive in most virus families, and is in fact more common than maintaining host specificity over evolutionary time. Understanding how viruses switch host specificities is critical for preventing or managing new viral outbreaks, but the evolutionary and molecular bases of virus host switching remain largely obscure. Keir proposes applying the extensive molecular toolkits developed for zebrafish and vaccinia virus research in a unique combination of model systems to identify host and viral determinants of virus host switching in novel cell culture and whole-animal settings. Poxviruses have been identified in fish, but the host range of vaccinia virus is restricted to mammals. He will identify virus determinants of host switching by adapting vaccinia virus to replicate in zebrafish. In parallel, he will characterize the evolution and function of the vertebrate interferon response in zebrafish to identify host determinants of virus replication and host switching. Given the constant threat of emerging virus infections, this work is poised to uncover molecular and evolutionary signatures that may improve our ability to monitor viral outbreaks in humans as well as fish and other agriculturally important vertebrate hosts. All animal studies will be performed following guidelines and protocols approved by the Institutional Animal Care and Use Committee of the University of Utah.

**Sierra Beach** (Department of Biological Sciences, University of Idaho, Moscow, ID, USA) presented empirical data based on computational modeling predictions of point mutations in the fusion glycoprotein (F protein) of respiratory syncytial virus (RSV) that may escape from monoclonal antibodies. Human RSV is the second leading cause of infant mortality in the world and is responsible for over 100,000 deaths each year. There is currently no licensed vaccine and the only treatment is palivizumab, a prophylactic monoclonal antibody, that targets the viral F protein. Given the lack of therapeutic options, mutations in the F protein that allow the virus to escape antibody neutralization are of high concern. Her lab seeks to establish a molecular model to predict antibody escape mutants in the F protein. Molecular dynamic simulations of the *F* gene were performed at the binding site of motavizumab, a palivizumab derivative. The predicted antibody escape mutations were engineered into an infectious clone for testing viability, fitness, and neutralization. In a parallel study, the wild type infectious clone was subjected to selective passages in vitro in the presence of motavizumab or the antibody AM14, to compare predictions to the variants isolated under selective pressure. Following selective passaging, samples that required higher antibody concentration for neutralization were sequenced and compared to predicted mutations. One predicted and one unpredicted *F* gene mutation were found in the AM14 samples and one predicted mutation was found in the motavizumab samples. While the results are promising, further experiments are needed to test the limits of the model. This study seeks to capture the powerful predictive capability of molecular modeling and transform it into clinically relevant watchlist and potential new therapeutics, setting the foundation for predicting mutations in other important viruses. Research funded by the National Science Foundation EPSCoR Research Infrastructure Improvement Program: Track-2, award number OIA-1736253. No animal or human studies were performed.

**Della Fixsen** (Elde Lab, University of Utah, Department of Human Genetics, Salt Lake City, UT, USA) presented work describing how poxviruses acquire host genes through active retrotransposition in infected cells. Horizontal gene transfer (HGT) provides a major source of genetic variation in virus evolution. Many viruses encode host proteins that have diverged and gained new functions altering cellular processes or subverting host defenses. While host gene acquisition is a common means of viral adaptation, mechanisms and dynamics of the process are not well understood. They devised a system to induce and select for HGT events in poxvirus populations, using vaccinia as a model. Mutant viruses lacking both *K3L* and *E3L* were propagated in complementing cells with *K3L* chromosomally integrated. They then screened for strains that overcame the antiviral PKR pathway to replicate in non-complementing cells. They recovered 10 virus clones from these experiments. Each strain independently acquired *K3L* via HGT from the complementing cells. Genomic analysis revealed target site duplications, poly(A) tails, and a lack of introns from the integrated construct, which all support a mechanism where *K3L* was retrotransposed into the viral gene through LINE-1 activity in infected cells. They also found corroborating evidence of LINE-1 mediated HGT in published genomes of diverse viruses. These results reveal that LINE-1 mobilization of host genes to virus genomes might represent a primary mechanism in virus evolution. No human or animal studies were performed.

**Emmanuel Ijezie** (Department of Biological Sciences and Center for Modeling Complex Interactions, University of Idaho, Moscow, ID, USA) presented on how rhinovirus curtails disease severity in respiratory viral co-infections in mice. Patients suffering from viral respiratory disease are often infected with multiple unrelated viruses, known as co-infection; however, it is unclear how viral co-infections influence disease pathogenesis. Their lab previously established a mouse model of respiratory viral co-infection to answer this question. Mice were inoculated with a mild respiratory virus (rhinovirus, RV1B) two days before a virus that causes severe disease (influenza A virus, PR8). Co-infection with RV1B reduced the severity of PR8 infection, as determined by mortality, weight loss, and clinical signs of disease. Mouse lungs were collected on days 4, 7, and 10 post-infection for pathological analyses. Lung tissues were paraffin-embedded for staining and further analysis. Histology slides were stained with Masson’s trichrome stain and hematoxylin and eosin (H&E) stain to evaluate inflammation and tissue damage, and immunohistochemistry was used to show localization of virus and immune cells. These analyses showed that RV1B mediates an early immune response that is characterized by neutrophil recruitment, followed by viral clearance and tissue repair. Host–pathogen interactions during viral co-infection are still poorly researched and these results will likely have an important role in elucidating mechanisms necessary for reducing the severity of influenza infection during respiratory viral co-infection. All animal studies were performed following guidelines and protocols approved by the Institutional Animal Care and Use Committee of University of Idaho.

**Andres Rodriguez** (University of Idaho, Moscow, ID, USA) presented data describing differences between viral co-infection and single infections on mouse lung epithelial cells. Patients hospitalized with respiratory infections have often been shown to be infected with multiple viruses. Studies with co-infected mice have shown there is a difference in infection severity if the mice are singly or co-infected. Little information exists on how two different viruses interact in shared cell population, and whether an immune response or competitive inhibition limits growth of one of the viruses. This study observes differences in viral titer from lung epithelial cell populations infected by a single virus or co-infected by murine coronavirus and rhinovirus. The supernatant medium was collected every six hours post-infection for up to 48 h. To determine viral titers at each timepoint, a 50% tissue culture infective dose (TCID50 Assay) was used. Comparing viral titers between multiple time points will give insight into the difference between the viral growth of co-infection against single infection. These data will provide insight into how co-infections behave within a cell population. All animal studies were performed following guidelines and protocols approved by the Institutional Animal Care and Use Committee of The University of Idaho. This project was funded in part by the National Science Foundation REU Site award No. 1757826.

**Frances V. Scholz** (Department of Biological Sciences and Center for Modeling Complex Interactions, University of Idaho, Moscow, ID, USA) studies respiratory syncytial virus (RSV), which is an intracellular pathogen that infects people of all ages. RSV is responsible for many deaths each year and currently, there is no licensed vaccine. In an alternate form of therapy, monoclonal antibodies can be used to treat infection by neutralizing the virus. They want to investigate the ability of RSV to mutate under stress of a human monoclonal antibody, D25. They hypothesized that RSV will mutate under stress of a sub-inhibitory dose of D25 resulting in escape from neutralization. Molecular modeling done by their collaborators will also accurately predict these mutations. To test this, they introduced RSV to rounds of selection in the presence of D25 and allowed time for mutations to arise. After 10 rounds of selection in HEp-2 cells, viral mutants required significantly more antibody for neutralization. The mutants were sequenced for specific amino acid changes and compared to the modeled predictions done by their collaborators. These results will help to better understand how RSV evolves to escape neutralization. No animal or human studies were performed.

**Sarah Stonedahl** (Department of Neurology, University of Colorado, School of Medicine, Aurora, CO, USA) investigated the role of microglia in controlling viral growth and subsequent virus-induced mortality in mice infected with West Nile Virus (WNV). WNV is a neurotropic, mosquito-borne, single-stranded RNA flavivirus. Eighty percent of human infections are asymptomatic, 20% of infections lead to an acute illness known as West Nile fever and less than 1% of human infections lead to severe neurological diseases such as encephalitis, meningitis, and acute flaccid paralysis. WNV infects neurons and causes cell death through caspase-3-dependent apoptosis. Previous work in their lab found that microglia (resident inflammatory cells of the brain) become activated and initiate an innate immune response to WNV infection. They also showed that microglia are critical during WNV CNS infection in mice for limiting viral growth and preventing mortality. However, the exact mechanism by which microglia protects the CNS remains unclear. To begin to investigate the role of microglia in limiting viral growth and mortality, they used in vivo (mouse) and ex vivo (brain slice cultures-BSCs) methods to model WNV encephalitis. They determined that while WNV is usually not seen in the brain of mice following peripheral infection until day 6 post infection, mice whose microglia had been depleted using the drug PLX5622 had measurable viral titers in the brain as early as day 4 post infection. It was also found using BSCs that there is an increase in caspase-3 activity in BSCs treated with PLX leading to the conclusion that microglia limit the amount of caspase-3 apoptosis in BSCs infected with WNV. All animal studies were performed following guidelines and protocols approved by the Institutional Animal Care and Use Committee of the University of Colorado School of Medicine.

**Joseph A. Westrich** (Department of Microbiology, Immunology, and Pathology, Colorado State University, Fort Collins, CO, USA) presented preliminary data on the establishment of an immune competent, small animal model of Zika virus infection. Approximately 80% of Zika virus (ZIKV) infections are subclinical. The recent ZIKV epidemic has revealed this mosquito-borne flavivirus as the causative agent of congenital abnormalities observed in babies born to ZIKV-infected mothers. ZIKV has been shown to cross the placental structure and infect the fetus, even in mothers with subclinical ZIKV infections. What remains poorly defined is the anti-viral immune response elicited by the presence of ZIKV at the maternal–fetal interface, and the impact it may have on pregnancy outcomes. Experimental models are urgently needed to recapitulate human disease. Many Zika pathogenesis studies have relied upon the use of immune deficient mice. These models, while having provided extensive insight to Zika infections, are unreliable to characterize the contribution of the immune response to ZIKV transplacental trafficking, fetal infection and potential life-long effects to offspring. Previous studies have shown that guinea pigs, an immune competent laboratory animal, are susceptible to ZIKV infection. Furthermore, the guinea pig is an appealing model for studies targeting investigation of the maternal-fetal interface because their placental structure and fetal development most closely resemble that of humans. Here they show preliminary data in the establishment of the guinea pig as a model for subclinical ZIKV infection. Guinea pig serological response to ZIKV and preliminary detection of viral genomes in peripheral blood suggest an active infection. Outwardly, the infected guinea pigs exhibited acute perturbations of temperature and weight loss, suggesting a subclinical infection. This immune competent small animal model will permit characterization of maternal ZIKV infection disease progression, anti-viral immune responses, and the consequence of these factors on fetal and offspring development. All animal studies were performed following guidelines and protocols approved by the Institutional Animal Care and Use Committee of Colorado State University.

### 2.4. Prevention and Treatment

**Chaoping Chen** (Department of Biochemistry and Molecular Biology, Colorado State University, Fort Collins, CO, USA) described HIV protease inhibitor susceptibility of protease autoprocessing as a correlative indicator of drug resistance. HIV-1 protease autoprocessing liberates the free mature protease from its Gag-Pol polyprotein precursor in concert with the virion release event. HIV protease inhibitors (PIs) suppress the mature protease activity through tight binding to the active site. While extensive sequence analysis has identified various major and minor resistance association mutations (RAMs) within the protease gene, recombinant mature PRs carrying these RAMs normally remain responsive to PI suppression, not correlating well with the clinical manifestation of PI resistance. They here report optimization and evaluation of a cell-based functional assay for quantification of autoprocessing efficiency and PI susceptibility using AlphaLISA (amplified luminescent proximity homogeneous assay ELISA). By analyzing a collection of 130 known protease inhibitors, the AlphaLISA assay confirmed that all 11 known anti-HIV protease inhibitors in the library suppressed precursor autoprocessing with low micromolar EC50s. The other protease inhibitors had no impact on precursor autoprocessing. Furthermore, autoprocessing of fusion precursors carrying various RAMs identified from patients experiencing PI resistance exhibited PI responsive profiles that closely matched to the reported PI resistance. They also found complex functional interplays among residues both inside and outside of the PR coding sequence attributed to modulation of autoprocessing activity and PI susceptibility in a context-dependent fashion. Collectively, their results support the idea that regulated precursor autoprocessing is a key determinant contributed to PI resistance development. Their AlphaLISA assay provides a powerful tool for identification and characterization of RAMs for their contribution to PI resistance and for correlative assessment of autoprocessing phenotype with clinic prognosis of PI responsiveness. No animal or human studies were performed.

**Brigette Corder** (University of Nebraska-Lincoln, Department of Biological Sciences, Lincoln, NE, USA) presented data comparing centralized genes used as a universal influenza vaccine. Each year Influenza A virus, an enveloped, negative sense, single-stranded, segmented RNA virus, causes millions of cases despite increased use of the annual flu vaccine. The current vaccination methods only protect against certain strains and, in the event of a mismatch virus, provide limited protection against infection and illness. For this reason, creating and testing a universal vaccine that induces broad protection against influenza is imperative. Here, they propose to explore centralized genes expressed in viral vectors as a promising influenza vaccine strategy for human influenza hemagglutinin 1 (H1). Hemagglutinin (HA) is a surface protein on influenza envelop which is involved in viral entry. Several centralized gene strategies will be tested which all seek to maximize the coverage of the population’s genetic divergence by analyzing all unique sequences in the Genbank database. Consensus HA are designed by using the most common amino acid at each location in the HA sequence. Mosaic HA are constructed by repeating in silico recombination events. Using a bias towards repeated 9-mer peptides, the mosaic strategy will favor repetitive 9-mers which will increase the number of potential B and T cell epitopes recognized by each HA. Each centralized HA gene will be expressed in pcDNA, recombinant replication-deficient adenovirus type 5 virus, and recombinant vaccinia virus. The cellular and humoral immune responses will be assessed and compared to wild-type genes expressed in the same viral vectors. Together, this project will reveal the most promising centralized antigen expressed in a viral vector for a universal influenza vaccine. All animal studies were performed following guidelines and protocols approved by the Institutional Animal Care and Use Committee of University of Nebraska Lincoln.

**Teresa Ng** (AbbVie Inc., North Chicago, IL, USA) presented an overview of the revolution in hepatitis C virus therapy. Chronic HCV infection is a major global health problem, with an estimated 70 million individuals infected worldwide. To date, seven distinct HCV genotypes have been identified. If undetected or left untreated, chronic HCV infection can lead to serious liver diseases such as cirrhosis, liver failure, and hepatocellular carcinoma. Sustained virologic response (SVR) resulting from treatment of chronic HCV infection has been shown to significantly reduce liver disease progression, development of hepatocellular carcinoma and mortality. The treatment of HCV infection has dramatically improved in the past decade. Pegylated interferon/ribavirin (pegIFN/RBV) has been available since early 2000, but this treatment has not been used widely due to its variable efficacy against different HCV genotypes (SVR ranging from ~50 to ~80%), severe side effects, long treatment durations (24 or 48 weeks), and the need to administer pegIFN by subcutaneous injection. The first HCV direct acting antivirals (DAAs), two different HCV NS3/4A protease inhibitors, were approved in 2011. Since then, a number of other HCV DAAs have been approved including inhibitors of HCV NS3/4A protease, NS5A protein, and NS5B polymerase. Current HCV treatment regimens are pegIFN-free, and include two or three DAAs targeting different HCV proteins, with and without the addition of RBV. The newest HCV DAA regimens approved in the past few years are convenient to take (once daily oral administration for as short as eight weeks), pegIFN- and RBV-free, efficacious in patients infected with any of the major HCV genotypes (SVR >95%), and exhibit a high barrier to the development of DAA resistance. The availability of these convenient and efficacious treatments has revolutionized efforts in the eradication of HCV infection globally. All studies using human subjects with AbbVie’s DAAs have been approved by the respective institutional review boards of the study sites.

### 2.5. RNA Viruses

**David Barton** (University of Colorado, School of Medicine, Aurora, CO, USA) described how viral RNA replication mechanisms impact error catastrophe. Asexual template-dependent RNA replication mechanisms, while efficient, render viruses susceptible to error catastrophe. Sexual RNA replication mechanisms, while inefficient, shape picornavirus species groups and counteract error catastrophe. They used viral RNA recombination assays, phylogenetic sequence data, and atomic structures of viral polymerases to identify features of the polymerase required for sexual RNA replication mechanisms. When they disabled sexual RNA replication mechanisms with a polymerase mutation, poliovirus becomes exquisitely sensitive to ribavirin-induced error catastrophe. Their data substantiate long held theories regarding the advantages and disadvantages of asexual and sexual replication strategies among RNA viruses. In particular, they showed that picornavirus RNA recombination counteracts the negative consequences of asexual template-dependent RNA replication mechanisms—namely error catastrophe. No animal or human studies were performed. Supported by NIH grants AI042189 & AI059130.

**Olve Peersen** (Colorado State University, Fort Collins, CO, USA) presented data from chimeric picornaviral polymerases built using the poliovirus and coxsackievirus B3 enzymes. The project foundation was prior structural biology work from the laboratory that showed a conserved polymerase fold analogous to a “right hand” composed of fingers, palm, and thumb domains, and the presence of a unique mechanism to close the active site for catalysis via a NTP-dependent conformation change in the palm domain. Biochemical studies of coxsackievirus B3 and poliovirus polymerases have showed that mutations in the palm domain result in drastic changes to both fidelity and elongation rate, with faster RdRPs having lower fidelity, leading to the hypothesis that the primary regulator of RdRP fidelity is the conformational change in the palm domain associated with active site closure. To further explore this, the lab generated chimeric polymerases that mix and match structural elements from the PV and CV enzymes. Eight chimeric polymerases were designed by swapping the entire fingers domain, just the “pinky” finger, or the thumb domain between PV and CV 3Dpol. The resulting polymerases were expressed in and purified from bacteria for biochemical assays of RNA binding and initiation, processive elongation rates, elongation complex stability, and nucleotide selectivity. In addition, the chimeric polymerases were inserted into infectious clones of both poliovirus and coxsackievirus B3, where a subset of them supported virus replication in cells. No animal or human studies were performed, and the work was supported by NIH award R01 AI-059130.

**Stephanie Moon** (Department of Biochemistry, University of Colorado, Boulder, CO, USA) presented work from a collaborative project with Tim Stasevich’s lab at Colorado State University on RNA-protein granules. Stress granules and P-bodies form during the integrated stress response when translation is globally suppressed. Both types of cytoplasmic assemblies contain diverse mRNAs and are enriched in RNA binding proteins, while stress granules harbor translation factors and P-bodies contain mRNA decay factors. Many RNA viruses including flaviviruses (e.g., West Nile virus and dengue viruses) inhibit stress granule formation despite causing global translation suppression of host mRNAs, suggesting the hypothesis that stress granules may have any anti-viral role in the cell. However, many of the fundamental properties and biological functions of stress granules are unknown. To gain insight into the biological role of stress granules during stress responses in regulating host cell gene expression, they applied nascent chain tracking to investigate the behavior of individual mRNA molecules during stress and simultaneously monitor mRNA translation and localization in human cells. Examination of the relationship between mRNA translation and stress granule formation revealed that most mRNAs that accumulate in stress granules are non-translating. In rare instances, mRNAs associated with nascent chains can dynamically interact with stress granules, but they do not observe mRNAs initiating translation while stably associated with stress granules. Furthermore, mRNAs can interact with stress granules in a transient or highly stable manner, with increased mRNA length and stress granule or P-body size corresponding with mRNA persistence within these mRNP granules. These results imply that the sequestration of mRNAs within stress granules in a stable state is a novel regulatory step dictating mRNA localization and function during stress. Future studies will examine the impact of viral RNA localization within stress granules on viral gene expression and replication. No human or animal studies were performed.

### 2.6. Prions

**Erin McNulty** (Prion Research Center, Colorado State University, Fort Collins, CO, USA), presented data describing how bioassay remains the gold standard for the detection of prion infectivity, and has provided tremendous insight to our understanding of prion pathogenesis and transmission dynamics. The conventional prion detection methods, IHC and western blot, have been heavily relied upon for their ability to detect PrPSc deposition in tissues of prion-infected hosts. In fact, IHC is considered the gold standard diagnostic test to demonstrate prion-infection. The recent introduction of assays employing amplification of prion seeds present in tissues and bodily fluids of infected hosts, sPMCA and RT-QuIC, have enhanced our ability to identify prions during the earlier phases of disease and in samples containing minute quantities of prions. This new generation of amplification assays have reported sensitivity levels rivaling that of bioassay. Thus, a variety of methodologies have been used to detect prions, yet to date, a cross platform comparison of these assays has not been performed. They compared conventional (IHC) and amplification assays (sPMCA and RT-QuIC) to determine their ability to detect prions in brain tissue harvested from a CWD mouse dilutional bioassay. They demonstrate that conventional IHC performs well in the detection of PrPSc deposition across the dilutional series. They also revealed enhanced detection of prion seeding activity in brain tissue at the lower end of the dilutional series if RT-QuIC is used as the readout for sPMCA. In summary, they provided evidence that the new generation of amplification assays enhance prion detection sensitivity and rigor over conventional assays. While in vitro detection methodologies will never replace bioassay, they may be used as tools to augment detection, alleviate the time and cost (financial and animal life) of animal bioassay, and become tools for the early diagnosis of human and animal protein misfolding disorders. All animal studies were performed following guidelines and protocols approved by the Institutional Animal Care and Use Committee of Colorado State University.

**Amy V. Nalls** (Department of Microbiology, Immunology, and Pathology, Colorado State University, Fort Collins, CO, USA) presented that chronic wasting disease (CWD) studies in the native white-tailed deer host—spanning 15 years—have provided a unique repository of serially collected samples. This repository has permitted the opportunity to evaluate intra- and inter-host CWD trafficking, dissemination, and transmission throughout the protracted disease course. The Mathiason lab has extended its previous studies to examine hematogenous prion load in blood collected minutes, days, weeks, and months post exposure, by employing enhanced amplification methodologies. They corroborated their previous findings by detecting amyloid formation in whole blood and blood cell fractions harvested as early as 15 minutes post inoculation, throughout the disease course, peaking at clinical disease. These findings bring the prion field a step closer to characterizing hematogenous prions and their role in the spread of prion disease. All animal studies were performed following guidelines and protocols approved by the Institutional Animal Care and Use Committee of Colorado State University.

**Kaitlyn Wagner** (Department of Microbiology, Immunology and Pathology, Colorado State University, Fort Collins, CO, USA) discussed studies aimed at detecting differences between chronic wasting disease strains from different states across the continental US. Chronic wasting disease (CWD) is a fatal prion disease affecting free-ranging and captive cervids. CWD was initially described in captive mule deer in the late 1960s. Since the initial description of CWD, it has been identified in 23 states, 2 Canadian providences, South Korea, and Norway. While some outbreaks of CWD were caused by transport of infected animals from endemic regions, the origin of CWD in other epizootics is unclear and has not been well characterized. Previous studies have shown that there are two distinct strains of CWD; however, the continuous spread and the unclear origin of several outbreaks warrant continued surveillance and further characterization of strain diversity. Additionally, a thorough understanding of prion strain diversity within ongoing outbreaks is lacking. To address these issues, they analyzed CWD-positive tissues from Michigan and Colorado to elucidate strain differences between and within different outbreaks. These preliminary analyses indicate that there is a strain difference between CWD in Colorado and CWD in Michigan. Additionally, there was no difference in CWD strain between the samples from Michigan, indicating that CWD-positive animals from MI are infected with a single CWD strain. Further studies will use transgenic mouse bioassay to further assess strain differences between these strains. Strain assessment from additional states is ongoing. The results from these investigations will improve our understanding of prion strain distribution, diversity, and evolution, as well as providing information that may prove useful to management strategies. No animal or human studies were performed.

**Julie Moreno** from the lab of **Glenn Telling** (Colorado State University, Prion Research Center and the Department of Microbiology, Immunology & Pathology, Fort Collins, CO, USA) presented information on transmissible spongiform encephalopathies (TSE); infectious neurodegenerative diseases caused by the misfolding of normal prion protein (PrPC) to (PrPSc). The only known cellular factor essential for prion propagation is PrPC; however, some cells, even with expression of PrPC, resist prion disease. Therefore, additional unidentified cellular factors regulate disease susceptibility and/or pathogenesis. To address this they compared the transcriptomes, using RNA sequencing (RNAseq), of cells that were susceptible (S) or resistant (R) to prion infection. These cells were derived from rabbit kidney epithelial (RK13) cells engineered to express PrP from deer, elk, and sheep and two mouse embryonic fibroblast (MEF) cell lines. Each S or R cell line was derived from the same parental clone indicating the S and R cells susceptibility difference was due to a genetic change. They compared S versus R RNA-seq raw counts from five cell lines and identified two differentially expressed genes using the DESeq normalization method. Transcripts from an oxidative stress-related gene (*Sps1*) decreased approximately two-fold in their S cells, while transcripts from a vesicle trafficking-related gene (*NBEAL1*) increased approximately 1.5-fold in S cells compared to R cells. These RNA-seq changes were verified using real-time PCR and protein expression techniques. They hypothesized that these candidate genes are susceptibility factors critical to the propagation of prions. NBEAL1 has been shown to be involved in vesicle trafficking and is highly expressed in the brain. Sps1 is involved in the recycling of selenocysteines (Sec), needed to further synthesize selenoproteins, many that are important in regulation of cellular redox homeostasis. Currently, they are undergoing analysis to determine the role of Sps1 and oxidative stress pathways in propagating infectious prions in their S and R cells using markers of reactive oxygen species (ROS) and determining levels of selenoproteins known to be involved in redox and oxidative stress pathways. Lastly, to determine if modulating levels of Sps1 and NBEAL1 alters prion propagation they are utilizing CRISPR/Cas9 gene editing techniques to remove the functional genes and an expression construct to increase levels in both S and R cells. Modulating these genes, in combination with the cervid prion cell assays (CPCA), will allow them to determine if Sps1 and/or NBEAL1 are critical cellular factors for prion propagation. No animal or human studies were performed.

### 2.7. Technology

**Ryan Fahy** (BioFrontiers Institute, Department of Molecular, Cellular, and Developmental Biology, University of Colorado, Boulder, CO, USA) explored the merits of implementing a targeted next-generation sequencing pipeline using the Illumina-Miseq platform instead of the traditional Sanger sequencing for examining a population’s host immune factors. Historically Sanger sequencing has been the gold standard for accurately sequencing individual genes and identifying mutations in genetic material. However, Sanger is not cost effective for sequencing a large number of samples and is unable to perform parallel investigation of multiple target regions. Targeted next-generation sequencing is able to produce a large amount of data for multiple target regions in high throughput applications relatively cheaply. This is ideal for investigating population-level diversity in many contexts. However, next-generation sequencing does have systematic biases that can result in base miscall and erroneous read alignment in regions of low complexity. Here, they compared these two strategies by gathering consensus sequences for 5 genes in 23 Ma’s Night Monkey (*Aotus nancymaae*) with both Sanger and the Illumnia Miseq platforms. They highlight the improved sensitivity that Illumina has for identifying SNP variants that were missed through Sanger sequencing due to allele-specific expression bias. At the same time, they show that this improved sensitivity of Illumina can be a problem when dealing with repetitive or paralogous gene families. These results underscore the importance of utilizing multiple platforms to test and develop next-generation sequencing pipelines that minimize the deficiencies of targeted next-generation sequencing while capitalizing on its strengths. All studies using human subjects or tissue samples have been either approved or deemed non-human subject research by the Institutional Review Board of The University of Colorado at Boulder.

